# Molecular Characterization of MYB28 Involved in Aliphatic Glucosinolate Biosynthesis in Chinese Kale (*Brassica oleracea* var. *alboglabra* Bailey)

**DOI:** 10.3389/fpls.2017.01083

**Published:** 2017-06-21

**Authors:** Ling Yin, Hancai Chen, Bihao Cao, Jianjun Lei, Guoju Chen

**Affiliations:** ^1^College of Horticulture, South China Agricultural UniversityGuangzhou, China; ^2^Vegetable Institute, Guangdong Academy of Agricultural SciencesGuangzhou, China

**Keywords:** Chinese kale, aliphatic glucosinolate, transcription factors, over-expression, RNAi

## Abstract

Glucosinolates are Brassicaceae-specific secondary metabolites that act as crop protectants, flavor precursors, and cancer-prevention agents, which shows strong evidences of anticarcinogentic, antioxidant, and antimicrobial activities. *MYB28*, the R2R3-MYB28 transcription factor, directly activates genes involved in aliphatic glucosinolate biosynthesis. In this study, the *MYB28* homology (*BoaMYB28*) was identified in Chinese kale (*Brassica oleracea* var. *alboglabra* Bailey). Analysis of the nucleotide sequence indicated that the cDNA of *BoaMYB28* was 1257 bp with an ORF of 1020 bp. The deduced BoaMYB28 protein was a polypeptide of 339 amino acid with a putative molecular mass of 38 kDa and a pI of 6.87. Sequence homology and phylogenetic analysis showed that *BoaMYB28* was most closely related to *MYB28* homologs from the *Brassicaceae* family. The expression levels of *BoaMYB28* varies across the tissues and developmental stages. *BoaMYB28* transcript levels were higher in leaves and stems compared with those in cotyledons, flowers, and siliques. *BoaMYB28* was expressed across all developmental leaf stages, with higher transcript accumulation in mature and inflorescence leaves. Over-expression and RNAi studies showed that *BoaMYB28* retains the basic *MYB28* gene function as a major transcriptional regulator of aliphatic glucosinolate pathway. The results indicated that over-expression and RNAi lines showed no visible difference on plant morphology. The contents of aliphatic glucosinolates and transcript levels of aliphatic glucosinolate biosynthesis genes increased in over-expression lines and decreased in RNAi lines. In over-expression lines, aliphatic glucosinolate contents were 1.5- to 3-fold higher than those in the wild-type, while expression levels of aliphatic glucosinolate biosynthesis genes were 1.5- to 4-fold higher than those in the wild-type. In contrast, the contents of aliphatic glucosinolates and transcript levels of aliphatic glucosinolate biosynthesis genes in RNAi lines were considerably lower than those in the wild-type. The results suggest that *BoaMYB28* has the potential to alter the aliphatic glucosinolates contents in Chinese kale at the genetic level.

## Introduction

Glucosinolates (GS) are nitrogen- and sulfur-rich plant amino acid-derived secondary metabolites found in *Arabidopsis thaliana* and the *Brassicaceae* family, such as broccoli, cabbage, cauliflower, kale, and turnip (Fahey et al., 1997; Kliebenstein et al., 2001; Wittstock and Halkier, 2002; Herr and Büchler, 2010). Glucosinolates play important roles in the plant defense system against insects and microbial infections (Manici et al., 1997). Glucosinolates and their hydrolytic products also contribute to the special flavors and odors of the *Brassicaceae* (Ishida et al., 2014). Based on the precursor amino acids used and R group modifications, glucosinolates are classified into three major chemical classes, namely aliphatic, aromatic, and indoic glucosinolates (Sønderby et al., 2010). Approximately 130 different aliphatic glucosinolates are known to occur in plants (Agerbirk and Olsen, 2012; Augustine and Bisht, 2015).

The aliphatic glucosinolate biosynthetic pathway has been elucidated in *A. thaliana*, including the genes responsible for side-chain elongation, core structure formation and secondary modification (Fahey et al., 2001; Grubb and Abel, 2006; Sønderby et al., 2010). MYB28, MYB29, and MYB76, which belong to subgroup-12 of the R2R3-MYB superfamily, act as direct transcriptional regulators of aliphatic glucosinolate biosynthesis (Gigolashvili et al., 2008; Baskar and Park, 2015). MYB28 has been shown to be the key regulator followed by MYB29, while MYB76 plays a minor and accessory role in the aliphatic glucosinolate pathway (Gigolashvili et al., 2009).

MYB28 positively regulates genes involved in aliphatic glucosinolate biosynthesis, including side-chain elongation (*MAM1* and *MAM3*) and core structure formation (*CYP79F1, CYP79F2, CYP83A1, ST5b*, and *ST5c*) (Gigolashvili et al., 2007; Augustine et al., 2013; Baskar and Park, 2015). In *A. thaliana*, AtMYB28 over-expression lines showed a significant increase in steady-state mRNA levels of aliphatic glucosinolate biosynthesis genes in leaves and inflorescences. In contrast, expression levels of aliphatic glucosinolate biosynthesis genes in AtMYB28 RNAi lines were lower than those in the wild-type (Gigolashvili et al., 2007). MYB28 controls the accumulation of long-chain and short-chain aliphatic glucosinolates (Chen et al., 2006; Hirai et al., 2007; Kim et al., 2013; Miao et al., 2013). In AtMYB28 over-expression lines, aliphatic glucosinolate contents were 2- to 7-fold higher than those in the wild-type, while glucoiberin and glucoalyssin levels were 2- to 3-fold higher than those in the wild-type. In contrast, in AtMYB28 RNAi lines, glucoraphanin, glucoiberin, and glucoalyssin levels were considerably lower than those in the wild-type. However, glucoerucin levels were neither increased nor decreased in over-expression or RNAi lines (Gigolashvili et al., 2007).

The expression pattern of MYB28 varies across tissues and developmental stages (Gigolashvili et al., 2007; Augustine et al., 2013; Baskar and Park, 2015). Expression levels of MYB28 in seedlings, stems, and siliques were higher than those in roots and primary leaf tissues of *Brassica juncea. Brassica nigra* showed a similar tissue-specific expression pattern (Augustine et al., 2013). In *Brassica rapa* ssp. *pekinensis*, MYB28 transcript levels are highest in inflorescences and stems (Kim et al., 2013). MYB28 is expressed throughout leaf development in *B. juncea*, with MYB28 expression being higher in mature and inflorescence leaves than in primary and young leaves (Augustine et al., 2013).

Chinese kale (*Brassica oleracea* var. *alboglabra* Bailey) is a rich source of antioxidants and anticarcinogenic compounds, including glucosinolates, carotenoids, vitamin C, and phenolic compounds (Choi et al., 2014). The market demand for Chinese kale is increasing due to its high nutritional value. Chinese kale is widely distributed in southern China, the Taiwan region, Japan, and Southeast Asia and has spread quickly to Europe and America (Sun et al., 2012a). Bolting stems are the most common edible parts of Chinese kale, as they are tender and crispy with good flavor. Consumption of tender rosette leaves and sprouts has also increased in southern China in recent years (Sun et al., 2011; Qian et al., 2015). In Chinese kale, the total glucosinolates contents varied extensively among the sprouts, rosette leaves and bolting stems, with highest content in the sprouts (Sun et al., 2011, 2012a,b). Compared with other glucosinolate, gluconapin was the most predominant glucosinolate in Chinese kale (Sun et al., 2011; Qian et al., 2015).

Although the role of MYB28 is very well known in other close relatives of the Chinese kale. The role and molecular characteristics of MYB28 in the aliphatic glucosinolate biosynthetic pathway of Chinese kale are unknown. Our study provides information on the molecular mechanisms of *BoaMYB28* in aliphatic glucosinolate biosynthesis in Chinese kale. The objectives of the study are to help breeders to develop cultivars with high beneficial aliphatic glucosinolate levels and low anti-nutritional aliphatic glucosinolates levels. The present study explores the molecular characterization of MYB28 in Chinese kale for the first time. Gene structure prediction, sequence alignment and phylogenetic analysis are investigated in detail. Differential expression profiles of BoaMYB28 have been carried out. To the best of our knowledge, this is the first report of the effect of *BoaMYB28* transformation on glucosinolate composition and content in *A. thaliana* and Chinese kale. We have also analyzed contents of aliphatic glucosinolate and expression levels of the aliphatic glucosinolate biosynthesis genes in *A. thaliana* and Chinese kale transgenic lines.

## Materials and Methods

### Plant Materials and Growth Conditions

Chinese kale seeds were germinated in plastic pots, and the seedlings were cultured in the field at 22–25°C at South China Agricultural University (Guangzhou, China). Bolting stems were harvested when plants were fully grown, with inflorescences as high as the apical leaves used for cDNA sequence cloning. For spatial expression pattern analysis, cotyledon (7 days), leaf (15 days), stem (30 days), flower (anthesis), and silique (15 days post-anthesis) were excised from 10 plants. For temporal expression pattern analysis, primary leaf (15 days), young leaf (30 days), mature leaf (60 days), and inflorescence leaf (anthesis) were excised from 10 plants.

*Arabidopsis thaliana* seeds (ecotype Col-0) were sterilized and plated on agar-solidified half-strength Murashige and Skoog (MS) medium containing 2.15 g/l MS salts and 0.5% sucrose, pH 5.8. Wild-type seeds were cold-treated at 4°C for 3 days in the dark and grown in a culture chamber at 21°C under 16/8 h light/dark cycle. Seedlings were transferred to soil and grown at 25°C under 16/8 h light/dark regime and at 40% humidity.

Plant materials were harvested in the early morning, weighed when fresh and washed with distilled water. All samples were immediately transported to the laboratory within 10 min, frozen in liquid nitrogen and lyophilised in an ultralow -80°C freezer for RNA isolation and/or for aliphatic glucosinolate content analysis.

### RNA Extraction and cDNA Synthesis

Total RNA was isolated using TRIzol reagent (Invitrogen, Carlsbad, CA, United States) followed by treatment with DNase (Promega, MA, United States) to remove possible DNA contamination. RNA quality and content were determined by gel electrophoresis and biophotometer (Eppendorf, Germany). First-strand cDNA was synthesized from 1 μg total RNA with an oligo-dT primer according to the instructions of the Reverse Transcriptase M-MLV Kit (Takara, Japan).

### Molecular Cloning of *BoaMYB28*

The full-length coding sequence of *BoaMYB28* was isolated from Chinese kale. The primers were designed based on a sequence identified in the BRAD *Brassica* database (Cheng et al., 2011). PCR amplification was performed using the primers listed in Supplementary Table S1. The PCR program was as follows: 95°C for 5 min; 35 cycles of 95°C for 1 min, 58°C for 1 min, and 72°C for 1 min; with a final extension at 72°C for 10 min. Five independent PCRs were used for isolating the gene. PCR products were ligated into the pMD18-T vector (Takara, Japan) and transformed into *Escherichia coli* DH5α. The *BoaMYB28* sequence was submitted to GenBank and was given the accession number KP723785.

### Expression Analysis of *BoaMYB28* and Aliphatic Glucosinolate Biosynthesis Genes

The expression patterns of the target genes were assayed by quantitative real-time PCR (qRT-PCR) using the primers listed in Supplementary Table S1. The reaction was performed using the MyiQ Real-Time PCR Detection System (Bio-Rad) platform using the SYBR Green Master Mix (Roche, Swiss) in a total 20 μl volume. The relative gene expression was calculated by the 2^-ΔΔCt^ method.

The amplification protocol was as follows: 95°C for 2 min; 35 cycles at 95°C for 15 s, 58°C for 30 s, and 72°C for 30 s; with a 5 min final extension at 72°C. β-actin was selected as the endogenous control gene for relative quantification. PCR reactions were performed from three independent biological replicates with three technical replicates for each. Data was analyzed using Bio-Rad CFX Manager software.

### Transformation of *BoaMYB28* in *A. thaliana* and Chinese Kale and Southern Blot

For generation of the *BoaMYB28* over-expression and RNAi construct, the coding sequence of *MYB28* was cloned into the plasmid vector, pFGC5941 (GenBank number AY310901) (Kerschen et al., 2004). The pFGC5941 vector contained the herbicide resistance gene (*bar*), which was used as selectable marker gene for generating transgenic lines (Supplementary Figures S10, S11). The different constructs were transformed into *Agrobacterium* strain GV3101 by the freeze-thaw method and further into *A. thaliana* by the floral dip method (Clough and Bent, 1998). Transgenic plants were selected on half-strength MS medium (0.5% sucrose, 0.8% agar, pH 5.8) (Gigolashvili et al., 2008).

Chinese kale seeds were surface sterilized with 70% ethanol for 5 min followed by 10% sodium hypochlorite for 10 min and then rinsed three times in sterile distilled water. The sterilized seeds were germinated for 1 week in half-strength MS medium with 30 g/l sucrose and 8 g/l agar at pH 5.8 (Murashige and Skoog, 1962). The hypocotyl explants were cultured for 3 days in MS medium with 1 mg/l 6-benzylaminopurine (BAP) and 1 mg/l naphthaleneacetic acid (NAA). The precultured hypocotyls were soaked in the overnight-cultured *Agrobacterium tumefaciens* strain EHA105 suspension for 5 min. The explants were cultured for 3 days in MS medium with the addition of 1 mg/l BAP and 1 mg/l NAA.

After washing any remaining bacteria away with sterile water, the explants were cultured for 1 week in MS medium with 500 mg/l cefotaxime sodium (Cef), 1 mg/l BAP, and 1 mg/l NAA. The explants were transferred to selective shoot regeneration medium containing MS basal medium with 5 mg/l phosphinothericin (ppt), 500 mg/l Cef, 1 mg/l BAP, and 1 mg/l NAA. After culturing for 2 weeks, the shoot buds were sub-cultured on MS medium with 10 mg/l ppt, 500 mg/l Cef, 1 mg/l BAP, and 1 mg/l NAA. The elongated green shoots were transferred to MS medium with 250 mg/l Cef and 2 mg/l NAA for rooting. After culturing for 4–5 weeks, plantlets with well-developed roots were hardened off and transplanted into the greenhouse for further analysis and recovery of seeds (Qian et al., 2015).

Southern blot was used to confirm that *MYB28* was integrated into *A. thaliana* and Chinese kale genomes (Zang et al., 2009). Aliquots of DNA (25 μg) were digested overnight with *EcoR*I, fractionated by 0.8% (w/v) agarose gel electrophoresis and transferred to a Hybond-N+ nylon membrane (Amersham Pharmacia Biotech, United Kingdom). The probe used for Southern analysis was *bar*, a selectable marker gene. Probe labeling, pre-hybridisation, hybridisation and detection were performed according to the protocol of DIG High Prime DNA Labeling and Detection Starter Kit II (Roche Diagnostics GmbH, Germany).

### Extraction and Determination of Glucosinolates in *A. thaliana* and Chinese Kale

Extraction and determination of aliphatic glucosinolates was performed in 5-week-old rosette leaves of transgenic and wild-type *A. thaliana*. Glucosinolates were extracted and analyzed as previously described (Gigolashvili et al., 2008). Freeze-dried samples were extracted twice with 70% methanol with the addition of glucotropaeolin (Applichem) as an internal standard. The methanol extracts were applied to a DEAE Sephadex A-25 column (acetic acid activated). The columns were sealed and desulphated overnight using purified *Helix pomatia* sulfatase (type H1, Sigma, Deisenhofen, Germany). HPLC analysis of desulphoglucosinolates was carried out as previously described (Gigolashvili et al., 2008). Detection of desulphoglucosinolates was performed at 229 nm by applying the response factors as described previously (Gigolashvili et al., 2007).

Glucosinolates of Chinese kale were extracted and analyzed as previously described (Sun et al., 2012a). The freeze-dried samples (500 mg) were mixed with 2 ml of 75% methanol at 80°C for 15 min and centrifuged at 10,000 × *g* for 5 min. The supernatant was further washed with 2 ml of 75% methanol and centrifuged at 10,000 × *g* for 5 min. The supernatant sample was applied onto DEAE-Sephadex A-25 column (acetic acid activated) and rinsed three times with 2 ml 0.02 M sodium acetate. The column was washed three times with 20 mM acetic acid and twice with water. The supernatant was mixed with 200 μl arylsulfatase solution. After incubation for 16 h at 35°C, the desulphoglucosinolates were eluted with 4 ml of Milli-Q water and filtered through a 0.45-μm membrane filter. HPLC analysis of desulphoglucosinolates was carried out as previously described (Qian et al., 2015). Glucosinolates were extracted using ortho-nitrophenyl-β-D-galactopyranoside (Sigma) as an internal standard.

### Bioinformatics Analysis

The amino acid sequence was predicted using DNAman. Multiple sequence alignments of amino acid sequences were performed using ClustalW. Phylogenetic trees were generated using MEGA based on the NJ (neighbor-joining) method. The confidence value for the phylogeny analysis was calculated using the bootstrap method. Molecular weight and isoelectric point of the protein were calculated with ProtParam (Brown et al., 2003). The conserved domains were predicted by CDD (Brown et al., 2003). The secondary structures and tertiary structures were predicted by SOPMA and SWISS MODEL (Brown et al., 2003). The transmembrane domains were predicted by TMHMM 2.0. The topology was determined using TMpred software. Signal peptide prediction was performed using SignalP 4.1. The hydrophilic and hydrophobic regions of the protein were predicted using ProtScale. Putative *N*-glycosylation sites were predicted using NetNGlyc 1.0 (Yu et al., 2004). Putative phosphorylation sites were predicted using NetPhos 2.0. YinOYang 1.2 Server was used to predict the putative *O*-glycosylation sites.

### Statistical Analysis

The data were analyzed with the SPSS software package version 11.5 (SPSS Inc. Chicago, IL, United States). Data were analyzed for statistical significance by one-way ANOVA model. The means were determined by the Least Significant Differences (LSD) test at *p* = 0.05.

## Results

### Molecular Cloning and Structural Analysis of *BoaMYB28*

We got the reference sequence from BRAD Brassica database. BRAD Brassica database is a new database which focuses on the genetics and genomics of the Brassica plants^1^. We designed the cloning primers by Primer 6.0 and determined the full-length of the target gene by PCR amplification. Two *MYB28* sequences (*MYB28-1* and *MYB28-2*) were cloned from different varieties of Chinese kale. The cDNAs of the two *MYB28* sequences were 98.57% identical with a total 18 different nucleotides over 1257 bp (Supplementary Figure S1). The deduced amino acid sequences were 100% identical (Supplementary Figure S2). We submitted only one sequence (*MYB28-1*) to GenBank (*BoaMYB28*). Analysis of the nucleotide sequence indicated that the *BoaMYB28* cDNA was 1257 bp with an ORF of 1020 bp (Supplementary Figure S3). The full-length cDNA contained a 138-bp 5′-untranslated region and 99-bp 3′-untranslated region. The deduced MYB28 protein sequence was 339 amino acids in length with a putative molecular mass of 38 kDa and a pI of 6.87. Homology searches against the Conserved Domain Database (CDD) revealed that *BoaMYB28* contained Myb-like DNA-binding domains (positions 14–61 and 67–112) and belonged to the SANT (SWI3, ADA2, N-CoR, and TFIIIB) superfamily. Secondary structure prediction by SDPMA showed that *BoaMYB28* contained 37.46% alpha helices, 45.72% random coils, 7.37% extended strand, and 9.44% beta turn (Supplementary Figure S4). A similar result was obtained after constructing the *BoaMYB28* three-dimensional structure using SWISS-MODEL (Supplementary Figure S5).

### Sequence Alignment and Phylogenetic Analysis of BoaMYB28

Alignment of the deduced amino acid sequence of BoaMYB28 revealed 73–98% identity with MYB28 proteins from 17 selected plant species. BoaMYB28 showed the higher level of sequence identity with MYB28 proteins from other members of the genus *Brassica* and lower levels of sequence identity with MYB28 proteins form other genera (**Table 1**).

**Table 1:** Amino acid sequence identities of Chinese kale *MYB28* with those of other species.

Organism	GenBank ID	Amino acid identity (%)
*Brassica oleracea* var. *italica*	GQ478992	98
*B. oleracea* var. *viridis*	AB702693	98
*B. napus*	KJ879593	98
*B. rapa* ssp. *pekinensis*	FJ584288	96
*B. rapa* ssp. *chinensis*	HQ270468	96
*B. nigra*	JX947842	93
*B. juncea*	JQ666167	93
*Thellungiella halophila*	AK353335	86
*Eutrema salsugineum*	XM_006394445	84
*Arabidopsis thaliana*	NM_125535	84
*A. lyrata* ssp. *lyrata*	XM_002866388	84
*Capsella rubella*	XM_006281649	83
*Camelina sativa*	XM_010485507	83
*Tarenaya hassleriana*	XM_010549468	83
*Malus* × *domestica*	XM_008352697	77
*Fragaria vesca* ssp. *vesca*	XM_004304237	75
*Prunus persica*	XM_007226528	73

**FIGURE 1:**
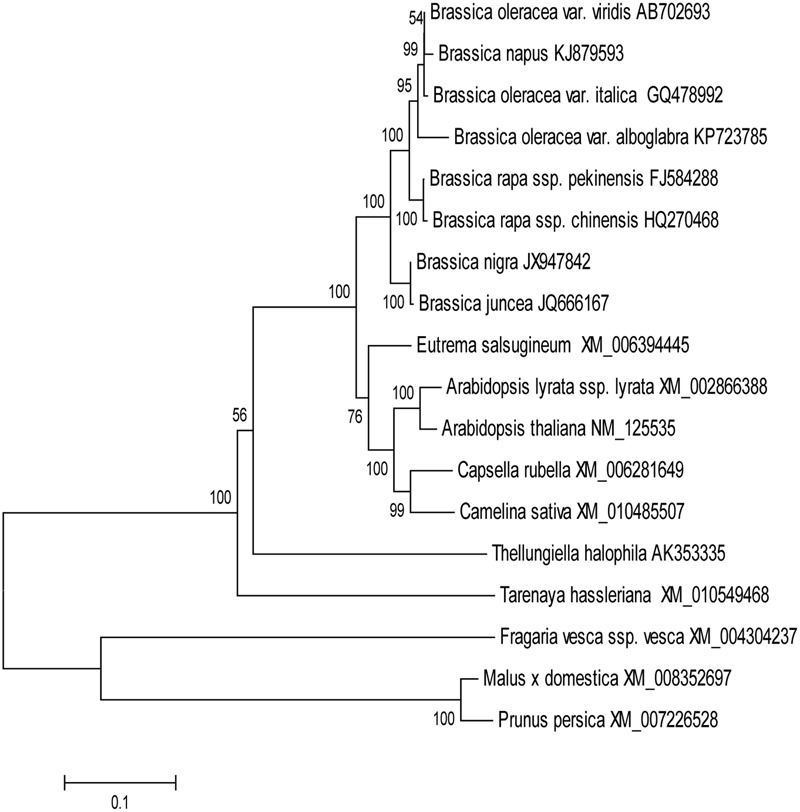
The phylogenetic tree of MYB28 proteins. The phylogenetic trees were generated based on the NJ (neighbor-joining) sequences distance method and constructed using MEGA 6.06 software. Numbers were bootstrap values for 1000 trails.

In order to evaluate the molecular evolutionary relationships, the NJ method was used to construct a phylogenetic tree of MYB28 protein sequences (**Figure 1**). The distances between different genera were relatively large while those within the same genus were relatively small. Multiple sequence alignment showed that BoaMYB28 was phylogenetically closer to MYB28 proteins from other *Brassica* vegetables than other genera. The phylogenetic relationships were in accordance with the classification and evolutionary status via morphological and biochemical characteristics.

### Structural Analysis of BoaMYB28

Bioinformatic tools were used to conduct structural analysis, such as identification of putative structural domains in BoaMYB28 proteins. The TMHMM results suggested that BoaMYB28 had no obvious transmembrane domain, which implied that BoaMYB28 was neither a membranous acceptor nor was located in the membrane. TMpred analysis predicted a lack of a transmembrane helix in BoaMYB28, and SignalP analysis also revealed a lack of a signal peptide. We examined the hydrophobic/hydrophilic nature of BoaMYB28 using ProtScale software, with a predicted maximum value of 1.5 and a minimum value of -3.578. Hydrophilic amino acid residues predominated in the peptide chain, indicating that BoaMYB28 might be a hydrophilic protein. NetNGlyc analysis identified three single *N*-glycosylation sites at positions 134, 177, and 276. Prediction of phosphorylation sites revealed 28 potential serine, 9 potential threonine, and 2 potential tyrosine targets were present in BoaMYB28. YinOYang analysis revealed that 12 potential *O*-glycosylation sites exist in BoaMYB28.

### Spatial and Temporal Expression Patterns of *BoaMYB28*

**FIGURE 2:**
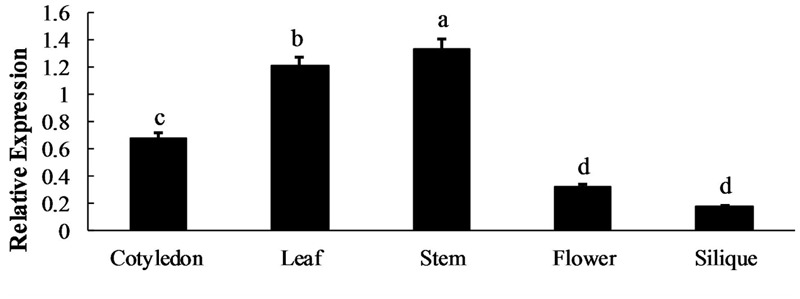
Spatial expression patterns of *BoaMYB28*. The tissues are defied as: cotyledon (7 days), leaf (15 days), stem (30 days), flower (anthesis), and silique (15 days post-anthesis). The data were analyzed using a one-way analysis of variance. β-actin mRNA expression was detected as the internal control. The same letters indicate no significant differences, while different letters indicate a statistically significant difference in expression. Each bar represents the mean (± standard error) of three independent biological replicates.

qRT-qPCR was used to analyze the BoaMYB28 mRNA expression levels in five tissues (cotyledon, leaf, stem, flower, and silique) and four developmental stages of leaf (primary leaf, young leaf, mature leaf, and inflorescence leaf). *BoaMYB28* was expressed in all of the collected tissues under normal growth conditions (**Figure 2**). However, *BoaMYB28* expression levels differed among the tissues. *BoaMYB28* was abundantly expressed in tissues that synthesize glucosinolates, such as leaves and stems, whereas cotyledons, flowers, and siliques showed lower transcript accumulation. *BoaMYB28* had the highest transcript abundance in stems and the lowest transcript accumulation in siliques. The expression level of *BoaMYB28* in stems was 9.52 times higher than that in siliques.

**FIGURE 3:**
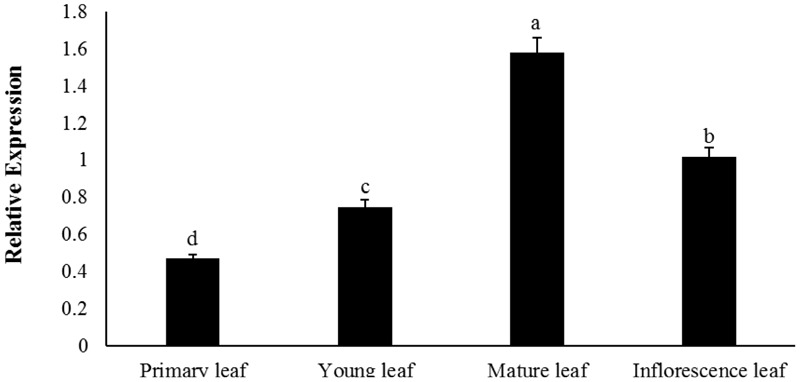
Temporal expression patterns of *BoaMYB28* at different developmental stages of leaves. The stages are defied as: primary leaf (15 days), young leaf (30 days), mature leaf (60 days), and inflorescence leaf (anthesis). The data were analyzed using a one-way analysis of variance. β-actin mRNA expression was detected as the internal control. The same letters indicate no significant differences, while different letters indicate a statistically significant difference in expression. Each bar represents the mean (± standard error) of three independent biological replicates.

The temporal expression of *BoaMYB28* was further determined at different developmental stages of Chinese kale leaves, tissues where glucosinolate synthesis predominantly occurs. During the developing stages of the leaves, *BoaMYB28* transcript levels were higher in the mature and inflorescence leaves than those in the primary and young leaves (**Figure 3**). *BoaMYB28* had the highest transcript abundance in the mature leaves and the lowest transcript abundance in the primary leaves. The expression level of *BoaMYB28* in the mature leaves was 4.2 times higher than that in the primary leaves.

### Glucosinolates Contents in Transgenic Plants

**FIGURE 4:**
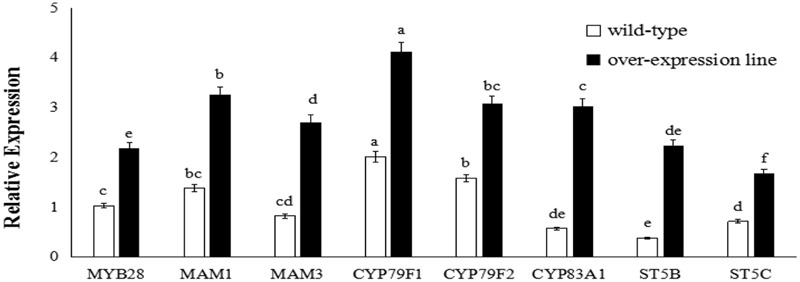
Transcript levels of aliphatic glucosinolate biosynthesis genes in *BoaMYB28* transgenic lines of *Arabidopsis thaliana*. Total RNA was prepared from rosette leaves of 5-week-old plants. Each PCR assay was repeated three times with two independent sets of plants. The data were analyzed using a one-way analysis of variance. β-actin mRNA expression was detected as the internal control. The same letters indicate no significant differences, while different letters indicate a statistically significant difference in expression. Each bar represents the mean (± standard error) of three independent biological replicates.

**FIGURE 5:**
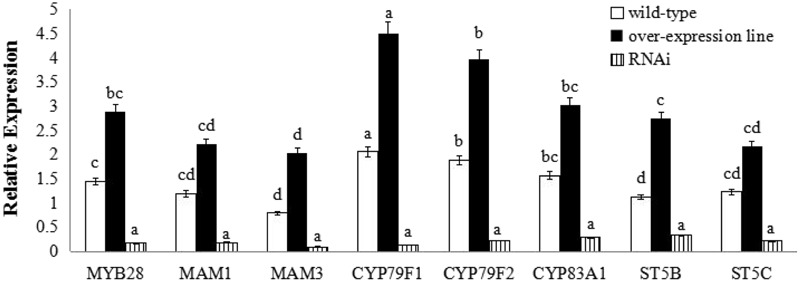
Transcript levels of aliphatic glucosinolate biosynthesis genes in *BoaMYB28* transgenic lines of Chinese kale. Total RNA was prepared from bolting stems. Each PCR assay was repeated three times with two independent sets of plants. The data were analyzed using a one-way analysis of variance. β-actin mRNA expression was detected as the internal control. The same letters indicate no significant differences, while different letters indicate a statistically significant difference in expression. Each bar represents the mean (± standard error) of three independent biological replicates.

In order to illustrate that aliphatic glucosinolates contents can be controlled by *BoaMYB28*, several transgenic over-expression and RNAi plants were created using *Agrobacterium tumefaciens*-mediated transformation. About 30 primary transformants were used for *BoaMYB28* over-expression constructs of *A. thaliana*. About 60 primary transformants were used for both *BoaMYB28* over-expression and RNAi constructs of Chinese kale. Three representative over-expressing lines (OE.Ara1, OE.Ara2, and OE.Ara3) of *A. thaliana* were chosen for further analysis. Three representative over-expressing lines (OE.Boa1, OE.Boa2, and OE.Boa3) and RNAi lines (RNAi.Boa1, RNAi.Boa2, and RNAi.Boa3) of Chinese kale were chosen for further analysis. T2 transformants were used for phenotypic and molecular analysis. Southern blot and qRT-PCR analysis confirmed that *BoaMYB28* had been integrated into *A. thaliana* and Chinese kale genomes (Supplementary Figures S6, S7 and **Figures 4, 5**). *BoaMYB28* transgenic lines of *A. thaliana* (OE.Ara1) and Chinese kale (OE.Boa1 and RNAi.Boa1) showed no visible differences in plant morphology (Supplementary Figures S8, S9).

Over-expression lines of *A. thaliana* exhibited increased levels of both short- and long-chain aliphatic glucosinolates compared to those of the wild-type (**Table 2**). Levels of 4-methylsulfinylbutyl-GS (4MSOB), the major aliphatic glucosinolate in *A. thaliana*, were found to be increased by 1.5- to 1.8-fold in over-expression lines. Levels of other short-chain aliphatic glucosinolates, such as 3-methylsulfinylpropyl-GS (3MSOP) and 5-methylsulfinylpentyl-GS (5MSOP), were increased by 1.7- to 2.3-fold. Accumulation of the long-chain aliphatic glucosinolate, 8-methylsulfinyloctyl-GS (8MSOO), also increased by 1.4- to 1.9-fold compared with that of the wild-type plants. The levels of indolic glucosinolates such as indolyl-3-methyl-GS (I3M), 1-methoxyindolyl-3-methyl-GS (1MOI3M), 4-methoxyindolyl-3-methyl-GS (4MOI3M), and 4-hydroxyindolyl-3-methyl-GS (4HOI3M), were found to be unaltered in the transgenic lines.

**Table 2:** The composition and content of glucosinolates (μmol⋅g^-1^ DW) in rosette leaves of *A. thaliana* among different transgenic lines and the wild-type.

	3MSOP	4MSOB	5MSOP	8MSOO	I3M	1MOI3M	4MOI3M	4HOI3M
WT	0.843^c^	4.908^d^	0.635^c^	0.372^c^	1.612^a^	0.597^a^	0.482^a^	0.173^a^
OE.Ara1	1.627^ab^	8.295^b^	1.346^ab^	0.597^b^	1.583^a^	0.581^a^	0.451^a^	0.154^a^
OE.Ara2	1.475^b^	7.706^c^	1.168^b^	0.625^ab^	1.557^a^	0.529^a^	0.503^a^	0.161^a^
OE.Ara3	1.881^a^	8.982^a^	1.416^a^	0.831^a^	1.639^a^	0.608^a^	0.491^a^	0.148^a^

*3MSOP, 3-methylsulfinylpropyl-GS; 4MSOB, 4-methylsulfinylbutyl-GS; 5MSOP, 5-methylsulfinylpentyl-GS; 8MSOO, 8-methylsulfinyloctyl-GS; I3M, indolyl-3-methyl-GS; 1MOI3M, 1-methoxyindolyl-3-methyl-GS; 4MOI3M, 4-methoxyindolyl-3-methyl-GS; 4HOI3M, 4-hydroxyindolyl-3-methyl-GS. OE.Ara1, OE.Ara2, and OE.Ara3 are three genetically stable T2 BoaMYB28 over-expression lines of *A. thaliana* WT refers to wild-type plants. Each value represents a mean (*n* = 3). Values in the same column followed by the same letter are not significantly different at *p* < 0.05.*

Levels of the main aliphatic glucosinolates, such as 2-hydroxy-3-butenyl-GS (2HO3BE), 2-propenyl-GS (2PE), 3-butenyl-GS (3BE), and 4MSOB were analyzed in the bolting stems of transgenic Chinese kale. The over-expression lines contained approximately 2- to 3-fold higher levels of 4MSOB compared to that of the wild-type (**Table 3**). Levels of 3BE, the alkenyl product of 4MSOB, were increased by 1.6- to 2.3-fold in over-expression lines. The level of 2HO3BE, the hydroxyl product of 3BE, was also increased by 1.9- to 2.6-fold. Accumulation of 2PE increased by 1.4- to 2.0-fold compared with that of the wild-type. The levels of 2HO3BE, 2PE, 3BE, and 4MSOB significantly decreased in the RNAi lines (**Table 3**). Levels of indolic glucosinolates such as I3M, 1MOI3M, 4MOI3M, and 4HOI3M were not affected in the transgenic lines.

**Table 3:** The composition and content of glucosinolates (μmol⋅g^-1^ DW) in bolting stems of Chinese kale among different transgenic lines and wild-type.

	2HO3BE	2PE	3BE	4MSOB	I3M	1MOI3M	4MOI3M	4HOI3M
WT	0.923^c^	1.218^c^	5.439^c^	0.745^c^	0.836^a^	0.417^a^	0.148^a^	0.354^a^
OE.Boa1	1.781^b^	1.744^c^	10.497^ab^	1.557^b^	0.821^a^	0.411^a^	0.152^a^	0.351^a^
OE.Boa2	2.169^ab^	2.497^a^	12.782^a^	2.287^a^	0.845^a^	0.432^a^	0.147^a^	0.363^a^
OE.Boa3	2.464^a^	2.156^ab^	9.083^b^	1.989^ab^	0.817^a^	0.398^a^	0.134^a^	0.349^a^
RNAi.Boa1	0.119^e^	0.228^d^	0.703^de^	0.189^e^	0.832^a^	0.402^a^	0.144^a^	0.342^a^
RNAi.Boa2	0.201^de^	0.242^d^	0.856^d^	0.307^d^	0.809^a^	0.423^a^	0.139^a^	0.338^a^
RNAi.Boa3	0.238^d^	0.143^e^	0.515^e^	0.282^d^	0.828^a^	0.396^a^	0.131^a^	0.352^a^

*2HO3BE, 2-hydroxy-3-butenyl-GS; 2PE, 2-propenyl-GS; 3BE, 3-butenyl-GS; 4MSOB, 4-methylsulfinylbutyl-GS; I3M, indolyl-3-methyl-GS; 1MOI3M, 1-methoxyindolyl-3-methyl-GS; 4MOI3M, 4-methoxyindolyl-3-methyl-GS; 4HOI3M, 4-hydroxyindolyl-3-methyl-GS. OE.Boa1, OE.Boa2, and OE.Boa3 are three genetically stable T2 BoaMYB28 over-expression lines of Chinese kale. RNAi.Boa1, RNAi.Boa2, and RNAi.Boa3 are three genetically stable T2 BoaMYB28 RNAi lines of Chinese kale. WT refers to wild-type plants. Each value represents a mean (*n* = 3). Values in the same column followed by the same letter are not significantly different at *p* < 0.05.*

### Expression Profiles of Glucosinolate Biosynthesis Genes in Transgenic Plants

*BoaMYB28* transgenic lines of *A. thaliana* (OE.Ara1) and Chinese kale (OE.Boa1 and RNAi.Boa1) were used for the qRT-PCR (**Figures 4, 5**). Increased transcript levels of genes involved in aliphatic glucosinolate biosynthesis corresponded well with the increased transcript level of *BoaMYB28* in the over-expression lines. In over-expression lines of *A. thaliana*, the expression levels of *MYB28*, side-chain elongation genes and core structure formation genes were 1.63-fold, 1.75- to 2.23-fold, and 1.39- to 4.02-fold higher than those in the wild-type. The expression levels of *MYB28*, side-chain elongation genes and core structure formation genes in over-expression lines of Chinese kale were 1.45-fold, 1.62- to 2.34-fold, and 1.21- to 3.18-fold higher than those in the wild-type. In contrast, a significant decrease in the expression levels of the target genes was observed in *BoaMYB28* RNAi lines. The results indicated that *BoaMYB28* was a positive regulator of aliphatic glucosinolate biosynthetic pathway genes in Chinese kale.

*BoaMYB28* up-regulated the aliphatic glucosinolate biosynthesis genes. *MAM1* and *MAM3* are involved in methionine chain elongation, the first phase of glucosinolate biosynthesis (Textor et al., 2007; Redovniković et al., 2012). *MAM1* catalyses the production of short-chained aliphatic glucosinolates, whereas *MAM3* is involved in the biosynthesis of long-chained aliphatic glucosinolates (Textor et al., 2007). The transcript levels of *MAM1* and *MAM3* in over-expression lines of *A. thaliana* were 1.82-fold and 2.05-fold higher than those in the wild-type, while the expression levels of *MAM1* and *MAM3* in over-expression lines of Chinese kale were 1.57-fold and 1.93-fold higher than those in the wild-type. However, the expression level of *MAM1* was higher than that of *MAM3* in over-expression lines of *A. thaliana* and Chinese kale. Although the expression level of *MAM1* was higher than that of *MAM3*, the change interval of *MAM3* was larger than *MAM1*. Consequently, *MAM3* was more tightly regulated by *BoaMYB28* compared with *MAM1.*

*CYP79F1* and *CYP79F2* are involved in biosynthesis of core structure of aliphatic glucosinolates (Chen et al., 2003). *CYP79F1* is responsible for the generation of short-chained aliphatic glucosinolates, while *CYP79F2* is capable of mediating the biosynthesis of long-chained aliphatic glucosinolates (Tantikanjana et al., 2004; Miao et al., 2013). In this study, *CYP79F1* and *CYP79F2* were differentially regulated by *BoaMYB28.* The transcript levels of *CYP79F1* and *CYP79F2* in over-expression lines of *A. thaliana* were 1.74-fold and 1.53-fold higher than those in the wild-type, while the expression levels of *CYP79F1 and CYP79F2* in over-expression lines of Chinese kale were 1.98-fold and 1.62-fold higher than those in the wild-type. The expression level of *CYP79F1* was higher than that of *CYP79F2* in over-expression lines of *A. thaliana* and Chinese kale. The results indicated that expression of *CYP79F1* was more tightly controlled by *BoaMYB28* compared with *CYP79F2*.

Other core structure formation genes (*CYB83A1, ST5B*, and *ST5C*) also showed the same and dramatic change in *BoaMYB28* transgenic lines of *A. thaliana* and Chinese kale, which was similar with the variation of *BoaMYB28.* The results indicated that *CYB83A1, ST5B*, and *ST5C* were positively regulated by *BoaMYB28.*

## Discussion

Glucosinolates and their hydrolytic products play important biological roles as flavor precursors, crop protectants and cancer-preventing compounds in cruciferous and other vegetables. The majority of genes and intermediates involved in aliphatic glucosinolate metabolism have been identified in *A. thaliana* (Hansen et al., 2001; Diebold et al., 2002; Gigolashvili et al., 2007, 2008; Li et al., 2013).

*MYB28* homologs have been isolated from *Brassica* crops such as *B. juncea, B. rapa*, and *B. nigra* (Augustine et al., 2013). In the present study, we identified *MYB28* for the first time in Chinese kale. Four MYB28 homologs (*BjuMYB28-1, -2, -3, -4*) have been isolated from *B. juncea* (Augustine et al., 2013), while three MYB28 homologs (*BrMYB28.1,.2,.3*) have been isolated from *B. rapa* ssp. *pekinensis* (Baskar and Park, 2015). In the current study, two *MYB28* sequences (*MYB28-1* and *MYB28-2*) were cloned from different varieties of Chinese kale. We submitted only one sequence of MYB28 (*MYB28-1*) to GenBank (*BoaMYB28*). It is necessary to further study whether if there are another member of MYB28 in Chinese kale.

In order to investigate the evolutionary origin further, an amino acid sequence alignment of MYB28 proteins from other plants was constructed. The amino acid sequence encoded by *BoaMYB28* shared close evolutionary ancestry with *MYB28* homologs from other *Brassica* plants. *BoaMYB28* exhibited a close genetic relationship with MYB28 proteins from other *Brassica* species. *B. oleracea* var. *italica, B. oleracea* var. *viridis* and Chinese kale (*B. oleracea* var. *alboglabra* Bailey) are all varieties of *B. oleracea*. *BoaMYB28* had the highest level of sequence identity with *B. oleracea* var. *italica* (98%) and *B. oleracea* var. *viridis* (98%). The results indicated that *MYB28* genes are evolutionarily conserved, retaining a very high level of sequence conservation while they have evolved via duplication (paralogs) and hybridisation (homologs) in two relatively simpler varieties of *B. oleracea*. The evolutionary relationships and classification of species can be approximated based on phylogenetic analysis (Ma et al., 2015). Phylogenetic analysis showed that *BoaMYB28* was most closely related to *MYB28* homologs from the *Brassicaceae* family, such as *B. oleracea* var. *italica* and *B. oleracea* var. *viridis*.

In *A. thaliana, MYB28* expression was detected mainly in generative organs and mature leaves (Gigolashvili et al., 2007). A similar observation was made in *B. nigra, B. juncea*, and *B. rapa* (Augustine et al., 2013). Expression levels of *BoaMYB28* were high in the commercial organs of Chinese kale, which are known for the accumulation of aliphatic glucosinolates. These results could facilitate tissue-specific engineering of improved aliphatic glucosinolate traits in Chinese kale. *MYB28* expression was high at the onset of the reproductive phase in *B. juncea*, which is a critical time of flowering and seed formation (Augustine et al., 2013). In *B. rapa* ssp. *pekinensis*, the expression level of *MYB28* was exclusively high in 2-month-old outer leaves (Baskar and Park, 2015). *BoaMYB28* was expressed throughout leaf development, with high transcript accumulation in the mature and inflorescence leaves. The temporal expression pattern of *BoaMYB28* suggested a role in aliphatic glucosinolate formation and distribution in Chinese kale leaves.

*BoaMYB28* has the same basic gene function as other *MYB28* genes that have been previously reported from other *Brassica* crops (Augustine et al., 2013). The unchanged growth phenotype of transgenic plants clearly demonstrated that *BoaMYB28* did not affect plant growth. Over-expression and RNAi of *BoaMYB28* in transgenic plants demonstrated that *BoaMYB28* positively regulated genes involved in the aliphatic glucosinolate biosynthetic pathway and controlled the accumulation of both short- and long-chain aliphatic glucosinolates. In over-expression lines of Chinese kale, the growth rates of expression levels of *CYP79F1* and *CYP79F2* were higher than those of *MAM1* and *MAM3*, which indicated that core structure formation genes was more tightly regulated by *BoaMYB28* compared with side-chain elongation genes in Chinese kale. Increases and decreases in aliphatic glucosinolate levels corresponded well with altered transcript levels of *BoaMYB28* in the transgenic lines. The altered aliphatic glucosinolate contents in transgenic lines of *A. thaliana* and Chinese kale correlated with altered mRNA levels of the genes involved in side-chain elongation (*MAM1* and *MAM3*) and core structure formation (*CYP79F1, CYP79F2, CYP83A1, ST5b*, and *ST5c*) of aliphatic glucosinolates. The levels of indolic glucosinolates remained unchanged in the transgenic lines, indicating that *BoaMYB28* specifically regulates aliphatic glucosinolate accumulation without affecting indolic glucosinolate levels.

## Conclusion

In conclusion, we have isolated the full-length cDNA of *BoaMYB28* from Chinese kale for the first time. Spatial and temporal gene expression patterns were detected, which may facilitate further studies on the regulatory metabolisms of aliphatic glucosinolate biosynthesis in Chinese kale. Aliphatic glucosinolate accumulation and expression profiles of aliphatic glucosinolate biosynthesis genes in transgenic lines of *A. thaliana* and Chinese kale were also investigated. This research results may be utilized to increase beneficial aliphatic glucosinolates and decrease anti-nutritional aliphatic glucosinolates for health benefits.

## Author Contributions

Designed the study: LY and JL. Provided the materials: HC, BC, and GC. Performed the experiment and analyzed the data: LY. Wrote the paper: LY and JL. All authors read and approved the final manuscript.

## Conflict of Interest Statement

The authors declare that the research was conducted in the absence of any commercial or financial relationships that could be construed as a potential conflict of interest.

## Footnotes

1 http://brassicadb.org/brad/
