# Supplementary material for: Molecular Characterization of MYB28 Involved in Aliphatic Glucosinolate Biosynthesis in Chinese Kale (Brassica oleracea var. alboglabra Bailey)
Source: Front Plant Sci. 2017 Jun 21;8:1083. doi: 10.3389/fpls.2017.01083 (PMC5478679; doi:10.3389/fpls.2017.01083)
Supplement: Supplementary file 1 [file Presentation_1.PDF]

Supplymentary Information

The title of the manuscript

Molecular characterization and expression analysis of MYB28 involved in aliphatic glucosinolates biosynthesis in Chinese kale (*Brassica oleracea* var. *alboglabra* Bailey)

The author list

Ling Yin[1, +], Changming Chen[1, +], Bihao Cao[1, +], Hancai Chen[2,], Jianjun Lei[1*,] and Guoju Chen[1, *]

[1] South China Agricultural University, College of Horticulture, Guangzhou, 510642, China

[2] Guangdong Academy of Agricultural Science, Vegetable Institute, Guangzhou, 510640, China

*corresponding. jjlei@scau.edu.cn, gjchen@scau.edu.cn

[+]these authors contributed equally to this work

TABLE S1| PCR Primers used in the study

Primers	Primer sequences (5′-3′)	Description
MYB28-F	GACCACCGAGGAAGATAAGAAAC	Sequence cloning, forward
MYB28-R	CCATGTCATGTATAAAATCAGCA	Sequence cloning, reverse
MYB28-F1	ATCCTATCAAAATTTACTTTCCTGC	Expression analysis, forward
MYB28-R1	CAGTCTACAACTCTTTCCACACCTT	Expression analysis, reverse
MAM1-F1	TTCCCACACTATCTTTCCTCCA	Expression analysis, forward
MAM1-R1	TACTCCGGCCACCGTTCCACAA	Expression analysis, reverse
MAM3-F1	TCTTCTTTTCCCTCTCTTCG	Expression analysis, forward
MAM3-R1	CTGCCTGGCAATCTCTAACT	Expression analysis, reverse
CYP79F1-F1	CATACCCTTTTCACATCCTACT	Expression analysis, forward
CYP79F1-R1	CTCGCTCTCTAAACGCTTCTCT	Expression analysis, reverse
CYP79F2-F1	TATCGTCTTCATCGCATCAATC	Expression analysis, forward
CYP79F2-R1	CCGTTTTTAGCTCTTTCATGGC	Expression analysis, reverse
CYP83A1-F1	CGGTTCTCCTTTTCTTCCTCT	Expression analysis, forward
CYP83A1-R1	TCACCACCATTGTTCTGCTTC	Expression analysis, reverse
ST5B-F1	TCAGAAACCCTAACCGCCAA	Expression analysis, forward
ST5B-R1	AGCCACCAGTAACCACCATA	Expression analysis, reverse
ST5C-F1	TTCAATGGAATCAGAAACCC	Expression analysis, forward
ST5C-R1	AACCTTCGAGGAGAGACGGT	Expression analysis, reverse
β-actin-F	TTGTTGGTAGGCCAAGACAT	Expression analysis, forward
β-actin-R	GGAGCTCGTTGTAGAAAGTG	Expression analysis, reverse

```
MYB28-1    TCTTATCTTATTAGAAAAAAAAAATCCTATCAAAATTTACTTTCCTGCAAGTATATTTTTCTTTACATTTT      70
MYB28-2    TCTTATCTTATTAGAAAAAAAAAATCCTATCAAAATTTACTTTCCTGCAAGTATATTTTTCTTTACATTTT      70

MYB28-1    CATTTTCTTGAGTGTTATTTGAGTGAAGTTATATTAAAATATTGTAATAGAGTTCATATATATCGAAAAT     140
MYB28-2    CATTTTCTTAAGTGTTATTTGAGTGAAGTTATATAAAAATATTGTGATGAAGTTCATATATATCGAAAAT     140

MYB28-1    GTCAAGAAAACCGTGTTGTGTCGGAGAAGGGCTGAAGAAAGGGGCATGGACCACCGAAGAAGATAAGAAA     210
MYB28-2    GTCAAGAAAACCGTGTTGTGTCGGAGAAGGGCTGAAGAAAGGGGCATGGACCACCGAAGAAGATAAGAAG     210

MYB28-1    CTCATCTCTTACATCCACGAACATGGAGAAGGAGGCTGGCGCGACATTCCCCAAAAAGCTGGATTGAAAA     280
MYB28-2    CTCATCTCTTACATCCACGAACATGGAGAAGGAGGATGGCGCGACATTCCCCAAAAAGCTGGATTGAAAA     280

MYB28-1    GGTGTGGAAAGAGTTGTAGACTGCGATGGACTAACTACCTAAAACCTGAGATCAAAAGAGGCGAGTTTAG     350
MYB28-2    GGTGTGGAAAGAGTTGTAGACTGCGATGGACTAACTACCTGAAACCTGAGATCAAAAGAGGCGAGTTTAG     350

MYB28-1    TTCAGAGGAGGAACAGATTATCATCATGCTTCATGCTTCTCGTGGAAACAAGTGGTCGGTCATAGCGAGA     420
MYB28-2    TTCAGAGGAGGAACAGATCATCATCATGCTTCATGCTTCTCGTGGAAACAAGTGGTCGGTCATAGCGAGA     420

MYB28-1    CATTTACCTAGAAGAACAGACAATGAGATCAAAAACTACTGGAACACACATCTCAAGAAGCGTTTGATTG     490
MYB28-2    CATTTACCTAGAAGAAGAGACAACGAGATCAAAAACTACTGGAACACACATCTCAAGAAGCGTTTGATTG     490

MYB28-1    AACAGGGTACTCATCCCCTGACTCACAAGCCACTAGCTTCTAATACAAACCCTACTGTACCTGAGAATTT     560
MYB28-2    AACAGGGTACTCATCCCCTGACTCACAAGCCACTAGCTTCTAATACAAACCCAACTGTACCTGAGAATTT     560

MYB28-1    GCATTCCCTAGATGCATCTAGTAATTCCGACAAGCAATACTCCCGGTCAAGCTCAATGCCTTCCATGTCT     630
MYB28-2    GCATTCCCTAGATGCATCTAGTAATTCCGACAAGCAATACTCCCGGTCAAGCTCAATGCCTTCCATGTCT     630

MYB28-1    TGTACTCCTTCCTCCGGTTTCAACACGGTTTTCGAGAATACCAGCAAAGATGGGACACCAGTTCGTGAGG     700
MYB28-2    TGTACTCCTTCCTCCGGTTTCAACACGGTTTTCGAGAATACCAGCAAAGATGGGACACCAGTTCGCGAGG     700

MYB28-1    ACGATTCCTTGAGTCGCAAGAAACGTTTGAAGAAATCAAGTTCTACATCAAGGCTTTTGAACAAAGTTGC     770
MYB28-2    ACGATTCCTTGAGTCGCAAGAAACGTTTGAAGAAATCAAGTTCTACATCAAGGCTTTTGAACAAAGTTGC     770

MYB28-1    GGCTAAGGCCACTTCCATGAAAGAAGCTTTGTCTGCTTCCATGGAAGGTAGCTTGAATGCTAATACAAGC     840
MYB28-2    GGCTAAGGCCACTTCCATGAAAGAAGCTTTGTCCGCTTCCATGGAAGGTAGCTTGAATGCTAATACAAGC     840

MYB28-1    TTTTCCAATGGCTACTCTGAGCAGATTCTCAATGAAGATGATAGTTCTAATGCATCCCTCATAAACACTC     910
MYB28-2    TTTTCCAATGGCTACTCTGAGCAGATTCTCAATGAAGATGATAGTTCTAATGCATCCCTCATAAATACTC     910

MYB28-1    TCGCCGAGTTCGATCCCTTCCTCCAAACAACGTTTTACCCTGAGAATGAGATGAATACTACTTCTGATCT     980
MYB28-2    TCGCCGAGTTCGATCCCTTCCTCCAAACAACGTTTTACCCTGAGAATGAGATGAATACTACTTCTGATCT     980

MYB28-1    CGGTATAGATCAGGACTACTTCTCACATTTTCTCGAAAATTTCGGCAGAGATGATGACCAGAATGAGGAG    1050
MYB28-2    CGGTATAGATCAGGACTACTTCTCACATTTTCTCGAAAATTTCGGCAGAGATGATGACCAGAATGAGGAG    1050

MYB28-1    CACTACATGAATCATAACTATGGTCATGATCTTCTTATGTCCGATGTGTTCCAAGAAGTCTCATCAACTA    1120
MYB28-2    CACTACATGAACCATAACTATGGTCATGATCTTCTTATGTCCGATGTGTTCCAAGAAGTCTCATCAACTA    1120

MYB28-1    GATCTTCATGCCCAAGCAGAAAGGTTTCAAACTTTTGAAACTTGTCAGAACAAGAAGTTATGTATGTATT    1190
MYB28-2    GATCATCATGCCCAAGCAGAAAGGTTTCAAACTTTTGAAACTTGTCAGAACAAGAAGTTATGTATGTTTT    1190

MYB28-1    CTCAAGCAGAAAGGTTTCAAACTTTTGAAACTTGTCAGAACAAGAAGTTATGTATGTATTCTATTA        1256
MYB28-2    CTCAAGCAGAAAGGTTTCAAACTTTTGAAACTTGTCAGAACAAGAAGTTATGTATGTATTCTATTA        1256
```

FIGURE S1| Comparison of nucleotide sequences of *MYB28-1* and *MYB28-2*.

```
MYB28-1    MSRKPCCVGEGLKKGAWTTEEDKKLISYIHEHGEGGWRDIPQKAGLKRCGKSCRLRWTNY     60
MYB28-2    MSRKPCCVGEGLKKGAWTTEEDKKLISYIHEHGEGGWRDIPQKAGLKRCGKSCRLRWTNY     60

MYB28-1    LKPEIKRGEFSSEEEQIIIMLHASRGNKWSVIARHLPRRTDNEIKNYWNTHLKKRLIEQG    120
MYB28-2    LKPEIKRGEFSSEEEQIIIMLHASRGNKWSVIARHLPRRTDNEIKNYWNTHLKKRLIEQG    120

MYB28-1    THPLTHKPLASNTNPTVPENLHSLDASSNSDKQYSRSSSMPSMSCTPSSGFNTVFENTSK    180
MYB28-2    THPLTHKPLASNTNPTVPENLHSLDASSNSDKQYSRSSSMPSMSCTPSSGFNTVFENTSK    180

MYB28-1    DGTPVREDDSLSRKKRLKKSSSTSRLLNKVAAKATSMKEALSASMEGSLNANTSFSNGYS    240
MYB28-2    DGTPVREDDSLSRKKRLKKSSSTSRLLNKVAAKATSMKEALSASMEGSLNANTSFSNGYS    240

MYB28-1    EQILNEDDSSNASLINTLAEFDPFLQTTFYPENEMNTTSDLGIDQDYFSHFLENFGRDDD    300
MYB28-2    EQILNEDDSSNASLINTLAEFDPFLQTTFYPENEMNTTSDLGIDQDYFSHFLENFGRDDD    300

MYB28-1    QNEEHYMNHNYGHDLLMSDVFQEVSSTRSSCPSRKVSN                          338
MYB28-2    QNEEHYMNHNYGHDLLMSDVFQEVSSTRSSCPSRKVSN                          338
```

FIGURE S2| Comparison of deduced amino acid sequences of *MYB28-1* and *MYB28-2*.

1	TCTTATCTTATTAGAAAAAAAAAATCCTATCAAAATTTACTTTCCTGCAAGTATATTTTTCTTTACATTT
70	TCATTTTCTTGAGTGTTATTTGAGTGAAGTTATATTAAAATATTGTAATAGAGTTCATATATATCGAAA
139	ATGTCAAGAAAACCGTGTTGTGTCGGAGAAGGGCTGAAGAAAGGGGCATGGACCACCGAAGAAGATAAG
1	M S R K P C C V G E G L K K G A W T T E E D K
208	AAACTCATCTCTTACATCCACGAACATGGAGAAGGAGGCTGGCGCGACATTCCCCAAAAAGCTGGATTG
24	K L I S Y I H E H G E G G W R D I P Q K A G L
277	AAAAGGTGTGGAAAGAGTTGTAGACTGCGATGGACTAACTACCTAAAACCTGAGATCAAAAGAGGCGAG
47	K R C G K S C R L R W T N Y L K P E I K R G E
346	TTTAGTTCAGAGGAGGAACAGATTATCATCATGCTTCATGCTTCTCGTGGAAACAAGTGGTCGGTCATA
70	F S S E E E Q I I I M L H A S R G N K W S V I
415	GCGAGACATTTACCTAGAAGAACAGACAATGAGATCAAAAACTACTGGAACACACATCTCAAGAAGCGT
93	A R H L P R R T D N E I K N Y W N T H L K K R
484	TTGATTGAACAGGGTACTCATCCCCTGACTCACAAGCCACTAGCTTCTAATACAAACCCTACTGTACCT
116	L I E Q G T H P L T H K P L A S N T N P T V P
553	GAGAATTTGCATTCCCTAGATGCATCTAGTAATTCCGACAAGCAATACTCCCGGTCAAGCTCAATGCCT
139	E N L H S L D A S S N S D K Q Y S R S S S M P
622	TCCATGTCTTGTACTCCTTCCTCCGGTTTCAACACGGTTTTCGAGAATACCAGCAAAGATGGGACACCA
162	S M S C T P S S G F N T V F E N T S K D G T P
691	GTTCGTGAGGACGATTCCTTGAGTCGCAAGAAACGTTTGAAGAAATCAAGTTCTACATCAAGGCTTTTG
185	V R E D D S L S R K K R L K K S S S T S R L L
760	AACAAAGTTGCGGCTAAGGCCACTTCCATGAAAGAAGCTTTGTCTGCTTCCATGGAAGGTAGCTTGAAT
208	N K V A A K A T S M K E A L S A S M E G S L N
829	GCTAATACAAGCTTTTCCAATGGCTACTCTGAGCAGATTCTCAATGAAGATGATAGTTCTAATGCATCC
231	A N T S F S N G Y S E Q I L N E D D S S N A S
898	CTCATAAACACTCTCGCCGAGTTCGATCCCTTCCTCCAAACAACGTTTTACCCTGAGAATGAGATGAAT
254	L I N T L A E F D P F L Q T T F Y P E N E M N
967	ACTACTTCTGATCTCGGTATAGATCAGGACTACTTCTCACATTTTCTCGAAAATTTCGGCAGAGATGAT
277	T T S D L G I D Q D Y F S H F L E N F G R D D
1036	GACCAGAATGAGGAGCACTACATGAATCATAACTATGGTCATGATCTTCTTATGTCCGATGTGTTCCAA
300	D Q N E E H Y M N H N Y G H D L L M S D V F Q
1105	GAAGTCTCATCAACTAGATCTTCATGCCCAAGCAGAAAGGTTTCAAACTTTTGAAACTTGTCAGAACAA
323	E V S S T R S S C P S R K V S N F *
1174	GAAGTTATGTATGTATTCTCAAGCAGAAAGGTTTCAAACTTTTGAAACTTGTCAGAACAAGAAGTTATG
1243	TATGTATTCTATTAT

FIGURE S3| Sequence analysis of nucleotide and deduced amino acid sequences of *BoaMYB28*.

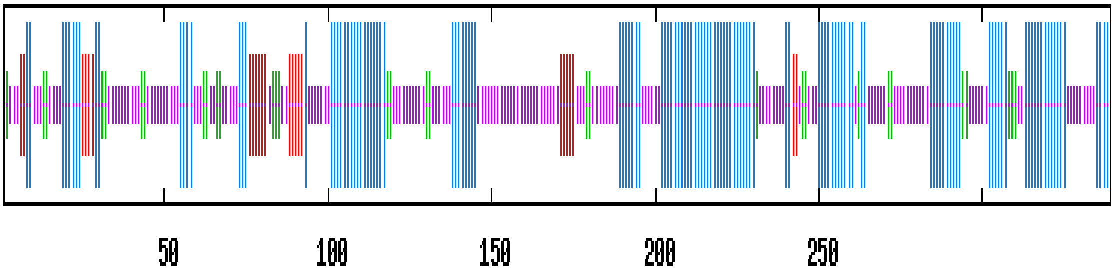

FIGURE S4| The secondary structure of BoaMYB28 generated by SOMPA. The longest vertical bar represents Alpha helix. The second longest one represents extended strand. The third longest one represents the random coil. The shortest one represents Beta turn.

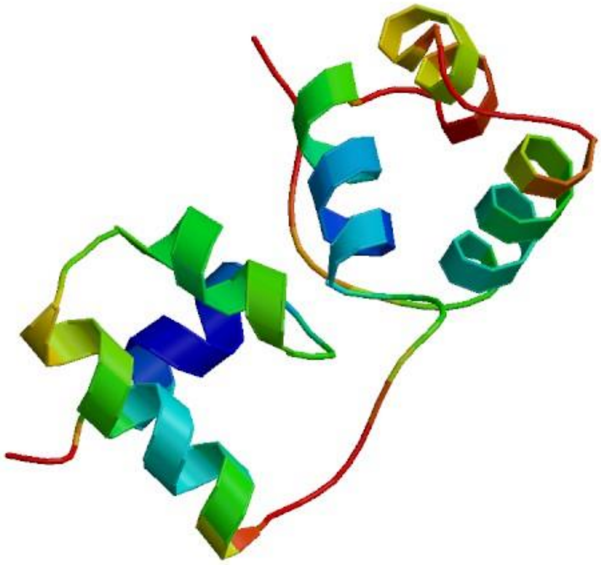

FIGURE S5| Three-dimensional model structure of BoaMYB28.

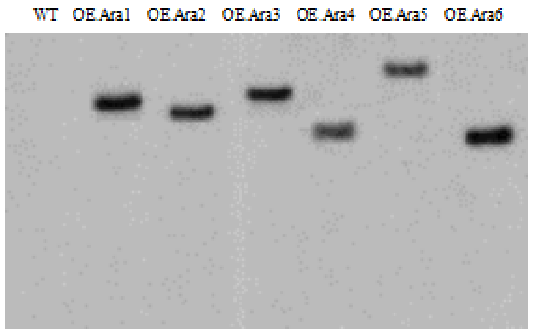

FIGURE S6| Southern blot analysis of *A. thaliana* among different transgenic lines and the wild-type. OE.Ara1, OE.Ara2, OE.Ara3, OE.Ara4, OE.Ara5 and OE.Ara6 are six genetically stable T2 BoaMYB28 over-expression lines of *A. thaliana*. WT refers to wild-type plants.

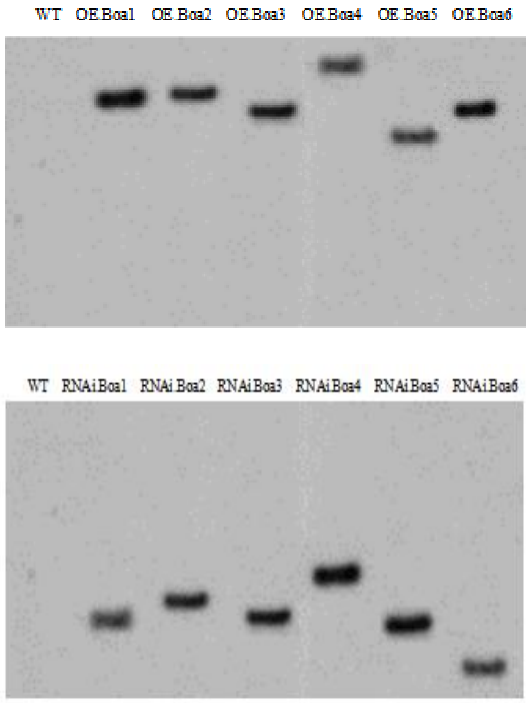

FIGURE S7| Southern blot analysis of Chinese kale among different transgenic lines and wild-type. OE.Boa1, OE.Boa2, OE.Boa3, OE.Boa4, OE.Boa5 and OE.Boa6 are six genetically stable T2 BoaMYB28 over-expression lines of Chinese kale. RNAi.Boa1, RNAi.Boa2, RNAi.Boa3, RNAi.Boa4, RNAi.Boa5 and RNAi.Boa6 are six genetically stable T2 BoaMYB28 RNAi lines. WT refers to wild-type plants.

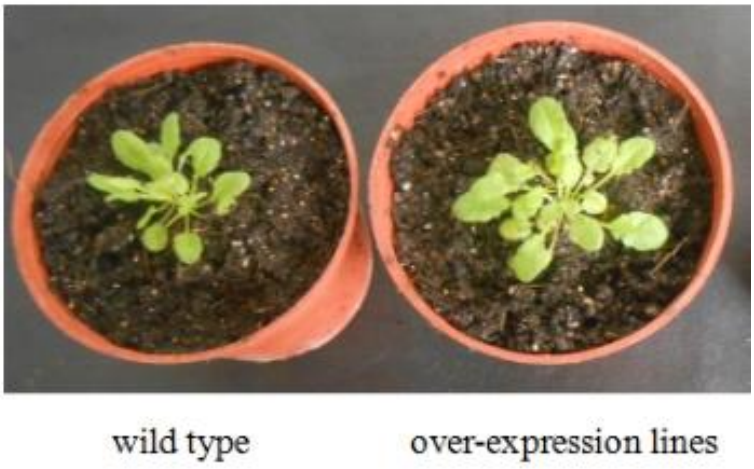

FIGURE S8| Growth phenotypes of *BoaMYB28* transgenic lines of *Arabidopsis thaliana*.

The transgenic lines are in the Col-0 wild-type background.

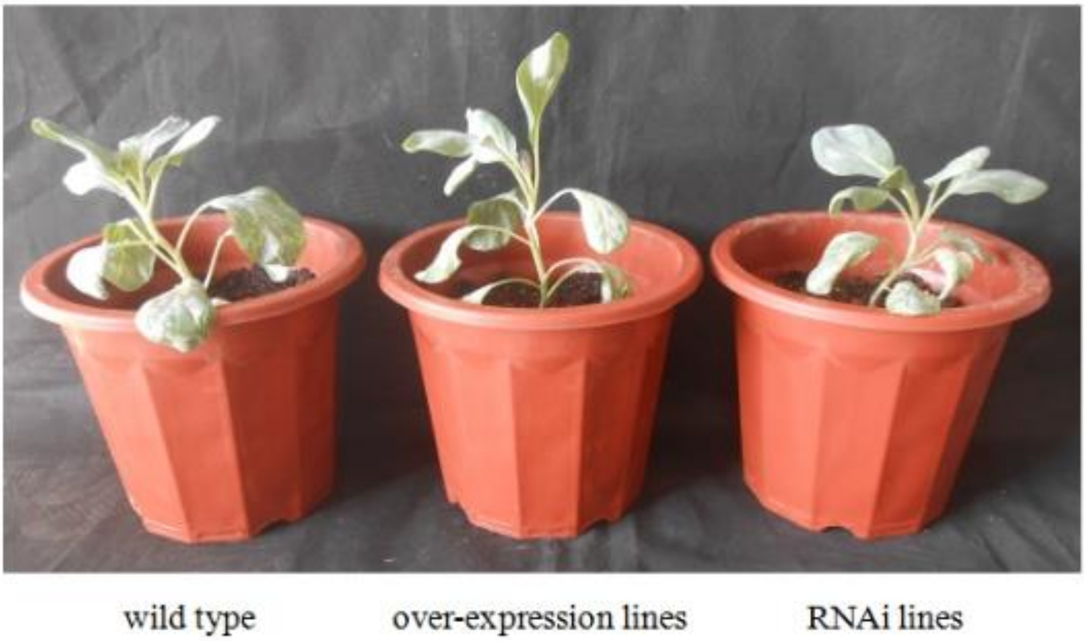

FIGURE S9| Growth phenotypes of *BoaMYB28* transgenic lines of Chinese kale.

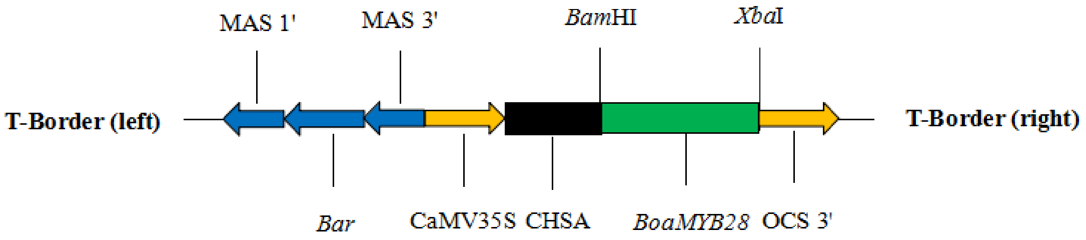

FIGURE S10| Schematic representation of the T-DNA region of *BoaMYB28* over-expression construct. *Bar*, herbicide resistance gene.

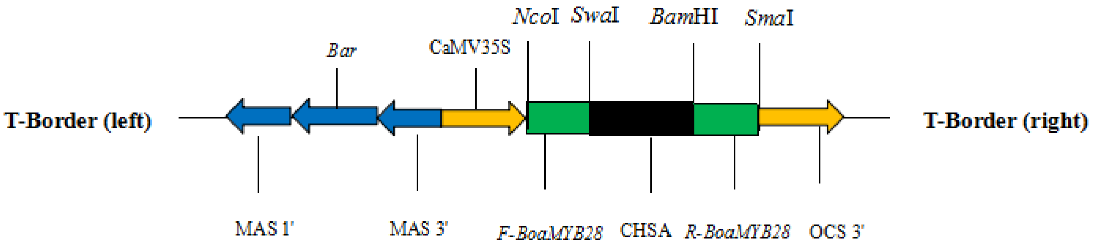

FIGURE S11| Schematic representation of the T-DNA region of *BoaMYB28* RNAi construct. *Bar*, herbicide resistance gene. *F-BoaMYB28*, forward DNA fragment of the *BoaMYB28* cDNA. *R-BoaMYB28*, reverse DNA fragment of the *BoaMYB28* cDNA.
